# Tissue Expression of NLRP3, IL‐1β, and IL‐18 in Glomerular and Interstitial Inflammation Associated With Diabetic Nephropathy

**DOI:** 10.1155/mi/9937801

**Published:** 2026-08-06

**Authors:** Bruna de Freitas Oliveira, Liliane Silvano Araújo, Crislaine Aparecida da Silva, Laura Penna Rocha, Raíssa Bernardes da Silva, Marlene Antônia dos Reis, Rafael Obata Trevisan, Bruna Cunha Zaidan, Juliana Reis Machado

**Affiliations:** ^1^ Kidney Research Center, Department of Pathology, Genetics and Evolution, Institute of Biological and Natural Sciences, Federal University of Triângulo Mineiro, Uberaba, Minas Gerais, Brazil, uftm.edu.br

**Keywords:** diabetic nephropathy, interleukin-1β, interleukin-18, NLRP3 inflammasome, renal inflammation

## Abstract

Activation of the NLRP3 inflammasome and its downstream cytokines IL‐1β and IL‐18 has been increasingly associated with the inflammatory mechanisms underlying diabetic nephropathy (DN), the leading cause of chronic kidney disease and end‐stage renal disease worldwide. This study investigated the expression of these inflammatory mediators in renal tissue from patients with DN. Eighty renal biopsies from adult patients (≥18 years) with a histopathological diagnosis of DN and 22 control samples obtained from autopsies were analyzed. NLRP3, IL‐1β, and IL‐18 expression was assessed by immunohistochemistry, and semi‐quantitative analysis was performed to determine the proportion of immunostained cells in the glomerular and interstitial compartments. A significant increase in immunostaining for all three mediators was observed in mesangial cells, podocytes, endothelial cells, and the interstitium of DN samples compared with controls (*p* < 0.05). Positive and significant correlations were found between NLRP3 and IL‐1β in endothelial cells (*p* < 0.0001; rS = 0.4720) and in the interstitium (*p* = 0.0230; rS = 0.2540), as well as between NLRP3 and IL‐18 in podocytes (*p* = 0.0055; rS = 0.3074). Moreover, interstitial expression of NLRP3 and IL‐1β correlated positively with serum creatinine levels (*p* = 0.0015; rS = 0.3708; and *p* = 0.0029; rS = 0.3480, respectively), suggesting an association between inflammasome‐mediated inflammation and renal dysfunction. Collectively, these findings indicate that the NLRP3/IL‐1β/IL‐18 axis plays a central role in glomerular and interstitial injury in DN, supporting its potential as a prognostic biomarker and a promising therapeutic target for disease modulation.

## 1. Introduction

Diabetes mellitus (DM) has shown a continuous increase in global prevalence and represents a major public health challenge due to its metabolic, hemodynamic, immunological, and renal complications. Among these, diabetic nephropathy (DN) is particularly relevant as it remains the leading cause of chronic kidney disease and end‐stage renal disease worldwide [1, 2]. The condition affects approximately 30% of patients with type 1 DM and 40% of those with type 2 DM [3], resulting from persistent hyperglycemia‐induced metabolic and hemodynamic alterations that culminate in glomerular injury, glomerulosclerosis, and tubulointerstitial fibrosis [4]. Clinically, DN is characterized by glomerular hyperfiltration, progressive albuminuria, decline in glomerular filtration rate (GFR), and eventual kidney failure [5].

Renal biopsy, considered the gold standard for the diagnosis of glomerular diseases—including DN—enables the identification of histopathological patterns that guide individualized therapeutic strategies [6, 7]. The use of biopsy in early stages may improve diagnostic accuracy, expand therapeutic opportunities, and enhance the understanding of early renal dysfunction, contributing to stricter control of metabolic complications associated with diabetes [8].

The pathophysiology of DN involves a complex network of interrelated mechanisms. Among metabolic pathways, the hexosamine pathway increases TGF‐β expression and promotes extracellular matrix accumulation [9, 10], while the polyol pathway exacerbates osmotic stress, activates protein kinase C (PKC), and contributes to matrix deposition [10]. The interaction of advanced glycation end products (AGEs) with their receptors activates NF‐κB, enhancing the production of proinflammatory cytokines and adhesion molecules—including IL‐1, IL‐6, IL‐18, TNF‐α, ICAM‐1, VCAM‐1, ESAM, E‐selectin, and α‐actinin‐4 [2, 11]. PKC activation further induces endothelin‐1, VEGF, and angiotensin II, aggravating glomerular injury [4]. Hemodynamic factors such as activation of the renin–angiotensin–aldosterone system amplify oxidative stress, intraglomerular hypertension, and proteinuria, whereas SGLT2‐mediated hyperfiltration contributes to mitochondrial dysfunction and a decline in GFR [5].

Low‐grade inflammation constitutes another key axis of DN, characterized by elevated cytokines such as IL‐6, TNF‐α, TGF‐β, and IL‐18 [2, 12] and also by the participation of IL‐1, IL‐33, and IFN‐γ [6]. Macrophage recruitment mediated by ICAM‐1 and VCAM‐1 exacerbates glomerular and tubular injury, further promoting the release of profibrotic mediators such as TGF‐β and IL‐1β [11, 13, 14]. Complement activation and renal hypoxia intensify inflammation and fibrosis [11, 13], whereas the JAK–STAT pathway plays a central role in mediating inflammatory and fibrogenic processes driven by hyperglycemia and AGEs [2, 4].

Within this multifaceted inflammatory landscape, the NLRP3 inflammasome emerges as a key driver of sterile inflammation in DN. Triggered by reactive oxygen species (ROS) and mitochondrial dysfunction, NLRP3 activates caspase‐1 and promotes the maturation of the proinflammatory cytokines IL‐1β and IL‐18 [15, 16]. NLRP3 activation correlates strongly with glomerulosclerosis and tubulointerstitial fibrosis [17], and its inhibition has been associated with reduced progression of DN [18].

The cytokines IL‐1β and IL‐18, both processed through inflammasome‐dependent signaling, are closely associated with the characteristic glomerular lesions of DN. IL‐1β exacerbates inflammation, disrupts glomerular structures, and promotes fibrogenic responses [3], making it an attractive therapeutic target [19]. IL‐18 plays a central role in regulating adhesion molecules, increasing ICAM‐1, VCAM‐1, and E‐selectin expression in endothelial cells, promoting leukocyte recruitment, and contributing to renal fibrosis during DN development [20].

Given this context, integrated evaluation of NLRP3, IL‐1β, and IL‐18 expression may elucidate key inflammatory mechanisms underlying DN progression. Therefore, the present study quantified the expression of these inflammatory markers in renal biopsies from adult patients with DN, aiming to deepen the understanding of their role in the processes contributing to disease severity and progression.

## 2. Methods

### 2.1. Patients

A total of 80 renal biopsies from adult patients (≥18 years) with a histopathological diagnosis of DN were analyzed. All samples were obtained from the database of the Kidney Research Center of the Federal University of Triângulo Mineiro (CePRim/UFTM), Uberaba, Minas Gerais, Brazil, collected between 2006 and 2024. All patients with confirmed DM and DN were included, provided they had no overlapping glomerular diseases, systemic nephritides, active infections, or any condition that could interfere with the renal inflammatory profile.

The control group consisted of 22 renal samples obtained from autopsies of adult individuals (≥18 years) whose cause of death was not related to renal, inflammatory, or infectious diseases. This group was matched by age and sex with the DN groups. These samples were provided by the Department of Pathology of the University of São Paulo (USP), in Ribeirão Preto, Brazil, where autopsies are performed within a short postmortem interval (PMI) due to the high service demand and the existence of a dedicated on‐call system for this purpose, which minimizes autolysis and favors the use of this material not only for diagnostic purposes but also for research.

Morphological analysis of all renal samples was performed by a nephropathologist, and only specimens with preserved tissue integrity and without significant autolysis, glomerulosclerosis, interstitial fibrosis, or inflammatory infiltrate were included. The PMI was not consistently available due to the retrospective nature of the study; however, all samples were collected and processed according to standardized institutional protocols. To ensure comparability, both autopsy‐ and biopsy‐derived samples were processed using identical fixation, embedding, and staining protocols. In addition, quality control measures were applied during the immunohistochemical procedures, including the use of appropriate positive and negative controls.

The study protocol was approved by the Institutional Ethics Committee of the Federal University of Triângulo Mineiro (approval number: 3.001.006).

### 2.2. Immunohistochemistry

Immunohistochemistry was manually performed on reserve slides containing 2 μm‐thick paraffin‐embedded renal sections. Slides were deparaffinized, rehydrated, and subjected to antigen retrieval using Trilogy solution (1:20) in a Pascal pressure chamber for 30 min. After washing in PBS, endogenous peroxidase activity was blocked with Peroxidase Block (Novolink) for 30 min in a dark, humid chamber, followed by two additional PBS washes (5 min each). A protein block (Protein Block, Novolink) was then applied for 25 min.

Slides were incubated overnight at 4°C with primary antibodies diluted in PBS containing 2% BSA, following manufacturer specifications summarized in Table 1. The next morning, slides were washed in PBS (3 × 5 min) and incubated with the Post Primary reagent (Novolink) for 30 min, followed by two additional PBS washes (2 × 5 min). The polymer detection system (Polymer, Novolink) was applied for 30 min and washed again (2 × 5 min).

**Table 1:** Technical specifications of the immunohistochemistry assays.

Primary antibody	Supplier	Clone or catalog number	Dilution
Polyclonal anti‐NLRP3 antibody	Thermo Fisher	PA5‐79740	1:500
Monoclonal anti‐IL‐1β antibody	Bio SB	PI3706	1:100
Monoclonal anti‐IL‐18 antibody	Thermo Fisher	MA5‐47203	1:500

Chromogenic development was performed using DAB (Liquid DAB, Dako) for 2 min, followed by rinsing in running water. Slides were counterstained with Harris hematoxylin for 1 min, dehydrated in graded ethanol (70%–95%), cleared in xylene (I–III), and mounted with permanent medium and coverslips.

Appropriate positive controls were used and consistently demonstrated strong and diffuse staining, supporting the sensitivity and adequacy of the immunohistochemical protocol.

### 2.3. Semi‐Quantitative Analysis of In Situ Immunostaining

The entire available tissue area on each slide was evaluated by two observers: an experienced pathologist and a PhD student highly trained by the pathologist, both blinded to the clinical diagnosis. Before the analysis, both observers underwent a calibration session to standardize the evaluation criteria. Discrepant cases were jointly reviewed until consensus was reached. Positive immunostaining was defined as cytoplasmic brown coloration in mesangial cells, podocytes, glomerular endothelial cells, or interstitial compartment cells.

The percentage of immunopositive cells was estimated in at least 10 representative fields per slide at 400× magnification. Immunostaining intensity was categorized according to a semi‐quantitative scale adapted from Ferro [21] and Kim et al. [22], ranging from 0% to 89%. No samples exhibited a staining between 90% and 100% (Table 2).

**Table 2:** Immunostaining intensity categories.

Percentage	Staining classification
0%–1%	Absent staining
2%–10%	Discrete staining
11%–49%	Moderate staining
50%–89%	Strong staining
90%–100%	Very strong staining

### 2.4. Statistical Analysis

Data were organized using Microsoft Excel, and statistical analyses and graph construction were performed using GraphPad Prism version 5.0 (GraphPad Software). The normality of variables was assessed using the Kolmogorov–Smirnov test. Results are expressed as the median and minimum–maximum values. Comparisons between the two groups were performed using the Mann–Whitney test. *p*‐Values were adjusted for multiple comparisons using the Benjamini–Hochberg false discovery rate (FDR) method, and results were considered statistically significant when the FDR was below 0.05. Correlations were analyzed using the Spearman’s rank correlation coefficient (rS). Statistical significance was set at *p* < 0.05.

Bivariate correlation and multivariable linear regression analyses were performed to evaluate the association between tissue expression of NLRP3, IL‐18, and IL‐1β and renal dysfunction, measured by GFR, in patients with DN. Correlations were assessed using Pearson or Spearman tests according to variable distribution and data characteristics. Complete‐case linear regression models were constructed using GFR as the dependent variable, with adjustment for systemic arterial hypertension, diabetes duration, and 24 h proteinuria. Each marker was analyzed separately according to the renal compartment being evaluated. Analyses were performed using IBM SPSS software version 20.0 (IBM Corp., Armonk, NY, USA), adopting a significance level of 5% (*p* < 0.05). Detailed multivariable regression analyses are presented in Supporting Information Table S1.

## 3. Results

A total of 80 patients diagnosed with DN were included in the study. Of these, 43 were male (53.7%) and 37 were female (46.2%), with a mean age of 52.8 ± 11.9 years. Regarding diabetes type, 36 patients (45%) had type 2 DM, nine (11.2%) had type 1 DM, and 35 (43.7%) had no recorded information. Disease duration exceeded 10 years in 45 patients (56.2%), ranged from 1 to 10 years in 21 patients (26.2%), and was unknown in 14 cases (17.5%).

Systemic arterial hypertension was the most frequent comorbidity, affecting 61 patients (76.2%), while 10 (12.5%) had no hypertension and nine (11.2%) lacked recorded information. Mean laboratory parameters included proteinuria of 5.34 ± 4.45 g/24 h, serum creatinine of 3.03 ± 3.86 mg/dL, estimated GFR (eGFR) of 37.9 ± 26.7 mL/min/1.73 m^2^, and serum glucose of 163 ± 85.2 mg/dL. Histopathological classification of DN, based on Tervaert et al. [23], is presented in Table 3.

**Table 3:** Distribution of diabetic nephropathy classes based on the Tervaert classification.

Class (*n*)	Patients with diabetic nephropathy (*n* = 80)
I	1
IIa	6
IIb	3
III	43
IV	27

**Figure 1:**
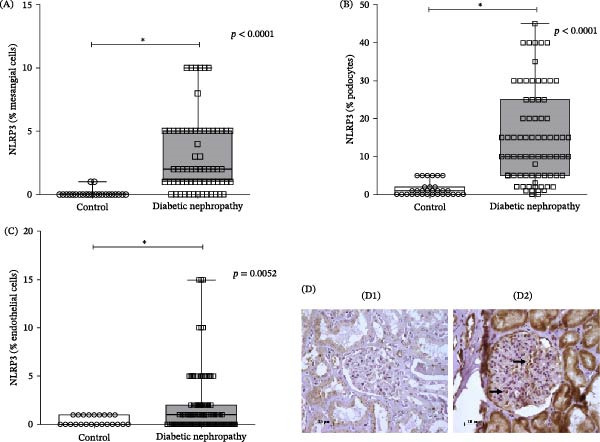
Expression of NLRP3 in glomerular compartments of control kidneys and diabetic nephropathy. (A) Mesangial cells (B) podocytes, and (C) endothelial cells. Boxplots represent median, interquartile range (25%–75%), and 10–90 percentiles. Mann–Whitney test;  ^∗^ *p* < 0.05. (D) Representative micrographs showing discrete NLRP3 staining in control glomeruli (D1) and strong immunostaining in mesangial cells and podocytes in diabetic nephropathy (D2), indicated by arrows.

Analysis of NLRP3 expression revealed a marked increase in the proportion of immunopositive cells in DN cases compared with that in controls. This increase was evident across glomerular compartments, including mesangial cells (Figure 1A; *p* < 0.0001), podocytes (Figure 1B; *p* < 0.0001), and glomerular endothelial cells (Figure 1C; *p* = 0.0052). Histological images showed discrete NLRP3 expression in control glomeruli, whereas DN biopsies exhibited intense staining in mesangial cells and podocytes (Figure 1D).

**Figure 2:**
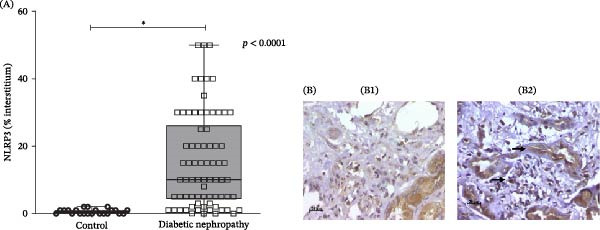
Expression of NLRP3 in the renal interstitium of control kidneys and diabetic nephropathy. (A) Percentage of NLRP3‐positive interstitial cells. Boxplots represent median, interquartile range (25%–75%), and 10–90 percentiles. Mann–Whitney test;  ^∗^ *p* < 0.05. (B) Representative micrographs showing absent or minimal NLRP3 staining in control interstitium (B1) and intense staining in inflammatory and tubular cells in diabetic nephropathy (B2), indicated by arrows.

A similar pattern was observed in the interstitial compartment, where NLRP3 expression was significantly higher in DN samples (Figure 2A; *p* < 0.0001), with micrographs demonstrating weak or absent staining in controls and strong labeling in inflammatory and tubulointerstitial epithelial cells in DN (Figure 2B). These findings highlight a consistent and robust upregulation of NLRP3 throughout glomerular and interstitial regions.

**Figure 3:**
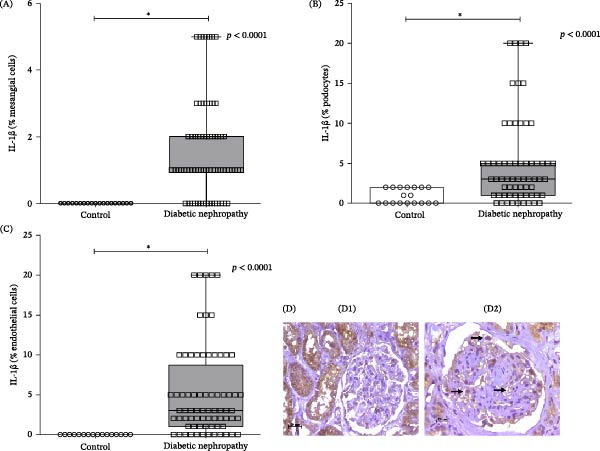
Expression of IL‐1β in glomerular compartments of control kidneys and diabetic nephropathy. (A) Mesangial cells, (B) podocytes, and (C) endothelial cells. Boxplots represent median, interquartile range (25%–75%), and 10–90 percentiles. Mann–Whitney test;  ^∗^ *p* < 0.05. (D) Representative micrographs showing weak or absent IL‐1β staining in control glomeruli (D1) and strong immunostaining in mesangial cells, podocytes, and endothelial cells in diabetic nephropathy (D2), indicated by arrows.

IL‐1β expression followed a similar trend, with significantly increased immunostaining in all renal compartments of DN patients. Within glomeruli, higher proportions of IL‐1β‐positive cells were observed in mesangial cells (Figure 3A; *p* < 0.0001), podocytes (Figure 3B; *p* = 0.0003), and endothelial cells (Figure 3C; *p* < 0.0001). Representative images depict minimal or absent IL‐1β staining in control glomeruli, in contrast to strong labeling in DN samples (Figure 3D).

**Figure 4:**
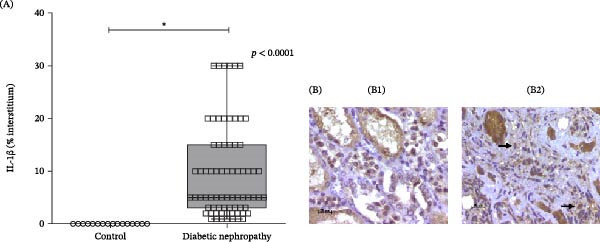
Expression of IL‐1β in the renal interstitium of control kidneys and diabetic nephropathy. (A) Percentage of IL‐1β‐positive interstitial cells. Boxplots represent median, interquartile range (25%–75%), and 10–90 percentiles. Mann–Whitney test;  ^∗^ *p* < 0.05. (B) Representative micrographs showing minimal or absent IL‐1β staining in control interstitium (B1) and intense immunostaining in inflammatory and tubular cells in diabetic nephropathy (B2), indicated by arrows.

In the interstitium, IL‐1β expression was also markedly elevated (Figure 4A; *p* < 0.0001), with histological sections showing negligible staining in controls and intense immunoreactivity in inflammatory and tubulointerstitial epithelial cells in DN (Figure 4B). Together, these data indicate that IL‐1β is broadly and consistently upregulated in DN, supporting its role as a key inflammatory mediator in renal injury.

**Figure 5:**
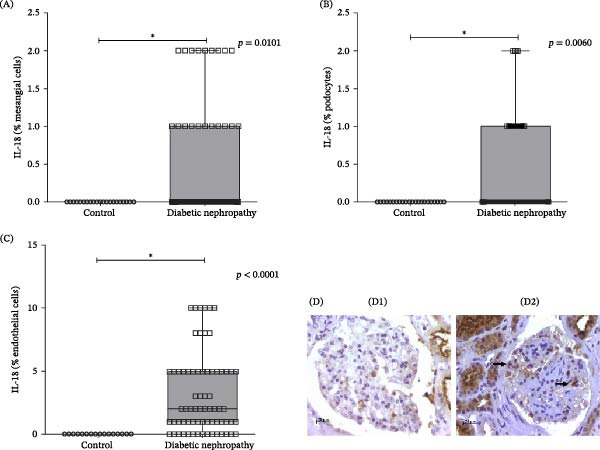
Expression of IL‐18 in glomerular compartments of control kidneys and diabetic nephropathy. (A) Mesangial cells; (B) Podocytes; (C) Endothelial cells. Boxplots represent median, interquartile range (25%–75%), and 10–90 percentiles. Mann–Whitney test;  ^∗^ *p* < 0.05. (D) Representative micrographs showing weak or absent IL‐18 staining in control glomeruli (D1) and strong immunostaining in podocytes, mesangial cells, and endothelial cells in diabetic nephropathy (D2), indicated by arrows.

IL‐18 expression was likewise significantly increased in DN biopsies. In glomeruli, elevated proportions of IL‐18‐positive cells were detected in mesangial cells (Figure 5A; *p* = 0.0181), podocytes (Figure 5B; *p* = 0.0046), and endothelial cells (Figure 5C; *p* < 0.0001). Histological images show weak or absent IL‐18 labeling in the control tissue, whereas DN glomeruli exhibited strong staining in multiple cell types (Figure 5D).

**Figure 6:**
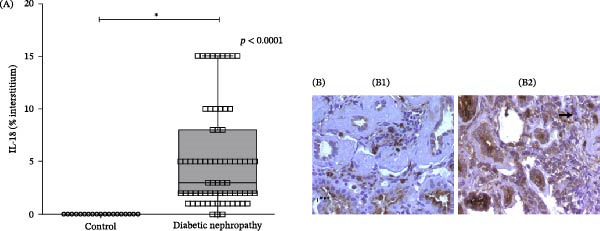
Expression of IL‐18 in the renal interstitium of control kidneys and diabetic nephropathy. (A) Percentage of IL‐18‐positive interstitial cells. Boxplots represent median, interquartile range (25%–75%), and 10–90 percentiles. Mann–Whitney test;  ^∗^ *p* < 0.05. (B) Representative micrographs showing minimal or absent IL‐18 staining in control interstitium (B1) and intense immunostaining in inflammatory and tubular epithelial cells in diabetic nephropathy (B2), indicated by arrows.

Interstitial IL‐18 expression was also significantly higher in DN cases (Figure 6A; *p* < 0.0001), with representative images illustrating the contrast between minimal staining in controls and marked immunoreactivity in inflammatory and epithelial cells in DN (Figure 6B). These findings reinforce the contribution of IL‐18 to both glomerular and interstitial inflammation in DN, consistent with inflammasome‐dependent cytokine activation.

All comparisons remained statistically significant after FDR correction (*q* < 0.05), confirming the robustness of the observed differences across the renal compartments.

**Figure 7:**
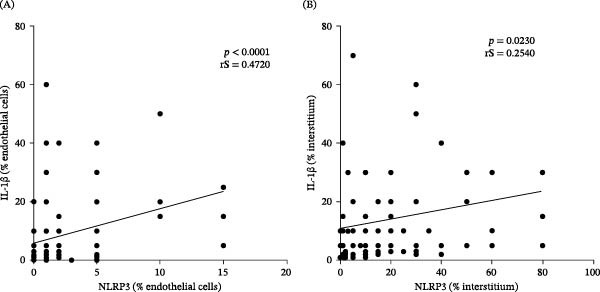
Spearman’s rank correlation between IL‐1β and NLRP3 expression in renal compartments. (A) Endothelial cells and (B) interstitium.

Given the upregulation of NLRP3, IL‐1β, and IL‐18, we next examined their relationships within the renal compartments. A significant positive correlation was observed between IL‐1β and NLRP3 expression in endothelial cells (Figure 7A; *p* < 0.0001; rS = 0.4720) and in the interstitium (Figure 7B; *p* = 0.0230; rS = 0.2540).

**Figure 8:**
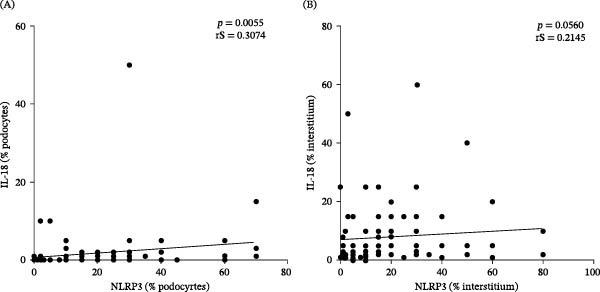
Spearman’s rank correlation between IL‐18 and NLRP3 expression in renal compartments. (A) Podocytes and (B) interstitium.

IL‐18 expression correlated positively with NLRP3 in podocytes (Figure 8A; *p* = 0.0055; rS = 0.3074), and a trend toward correlation was observed in the interstitial compartment (Figure 8B; *p* = 0.0560; rS = 0.2145). These patterns suggest cooperative interactions across multiple cell types in mediating renal inflammation in DN.

**Figure 9:**
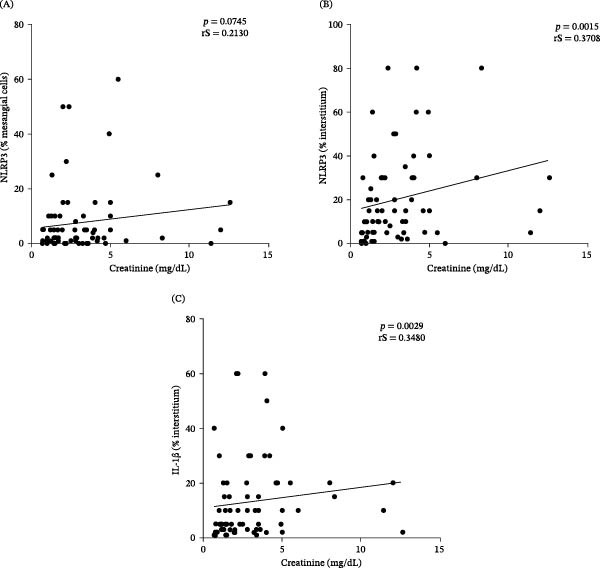
Spearman’s rank correlation between inflammatory markers and serum creatinine levels. (A) Correlation between NLRP3 expression in mesangial cells and serum creatinine. (B) Correlation between NLRP3 expression in interstitium and serum creatinine. (C) Correlation between IL‐1β expression in interstitium and serum creatinine.

Finally, correlations with clinical renal function revealed a trend toward a positive association between NLRP3 expression in mesangial cells and serum creatinine levels (Figure 9A; *p* = 0.0745; rS = 0.2130). In the interstitial compartment, both NLRP3 (Figure 9B; *p* = 0.0015; rS = 0.3708) and IL‐1β (Figure 9C; *p* = 0.0029; rS = 0.3480) exhibited significant positive correlations with serum creatinine, indicating that inflammasome‐related inflammation is closely linked to renal dysfunction in DN.

In bivariate analyses restricted to patients with DN (*n* = 70), the expression of NLRP3, IL‐18, and IL‐1β in the inflammatory infiltrate showed an inverse correlation with GFR (Table 4). The strongest correlation was observed for NLRP3 expression in the infiltrate, although the coefficients indicated weak‐to‐moderate associations.

**Table 4:** Bivariate correlations between clinical parameters, inflammasome markers across renal compartments, and GFR in patients with diabetic nephropathy.

Variable	Compartment	Correlation coefficient	*p*‐Value	*N*
Inflammasome markers				
NLRP3 (%)	Podocytes	−0.240	0.046 ^∗^	70
	Infiltrate	−0.438	<0.001 ^∗∗∗^	70
IL‐18 (%)	Podocytes	0.025	0.834	70
	Infiltrate	−0.261	0.029 ^∗^	70
IL‐1β (%)	Podocytes	−0.020	0.870	70
	Infiltrate	−0.338	0.004 ^∗∗^	70
Clinical covariates				
Hypertension	Systemic	−0.142	0.272	62
Diabetes duration	Systemic	0.002	0.987	58
Proteinuria (mg/24 h)	Renal	0.019	0.880	63

*Note*: Correlation coefficients (Pearson’s *r* or Spearman’s rho) indicate the direction and magnitude of the association between the variables and GFR. Spearman’s rho was used for non‐normally distributed variables and binary/ordinal clinical covariates. Pearson’s *r* was used for normally distributed continuous variables. “*N*” denotes sample size. Statistical significance is denoted by asterisks:  ^∗^ *p* < 0.05;  ^∗∗^ *p* < 0.01;  ^∗∗∗^ *p* < 0.001.

Abbreviation: eGFR, estimated glomerular filtration rate.

To evaluate whether these associations persisted after adjustment for clinical covariates, multivariable linear regression models were performed using complete case analysis (*n* = 46), with GFR as the dependent variable. Each marker was evaluated in a separate model adjusted for systemic arterial hypertension, diabetes duration, and proteinuria. In these adjusted models, NLRP3 expression in the inflammatory infiltrate remained inversely associated with GFR (*B* = −53.131; 95% CI: −101.019 to −5.244; *p* = 0.031), as did IL‐1β expression in the infiltrate (*B* = −73.940; 95% CI: −140.658 to −7.223; *p* = 0.031). No significant adjusted associations were observed for glomerular/podocyte marker expression or for IL‐18.

## 4. Discussion

DN is one of the most relevant and devastating complications of DM, and the understanding of its pathophysiological mechanisms has advanced substantially over the recent decades. Although traditionally described as a consequence of chronic hyperglycemia–induced metabolic and hemodynamic alterations, emerging evidence demonstrates that sterile inflammation and innate immunity may play important roles in the initiation and progression of renal injury. The findings of this study are consistent with this concept, showing a significant increase in the expression of NLRP3, IL‐1β, and IL‐18 across multiple renal compartments, suggesting both systemic and compartmentalized activation of the inflammasome in DN.

The strong immunostaining observed in mesangial cells, podocytes, endothelial cells, and the interstitial compartment suggests that inflammasome activation may not be restricted to a single cell type or disease stage. Rather than demonstrating, our data are compatible with a sustained inflammatory axis permeating the distinct phases of DN pathophysiology. Mesangial cells, for instance, respond robustly to inflammatory stimuli derived from AGEs, oxidative stress, and mechanical changes induced by hyperfiltration, promoting NLRP3 activation and potentially contributing to mesangial expansion, extracellular matrix deposition, and glomerulosclerosis. This response aligns with studies showing heightened mesangial sensitivity to angiotensin II, ROS, and NF‐κB under hyperglycemic conditions, all of which are potential inducers of the NLRP3 pathway [24].

The pronounced expression of NLRP3, IL‐1β, and IL‐18 in podocytes is particularly noteworthy given the essential role of these cells in maintaining the glomerular filtration barrier. Podocyte dysfunction is considered one of the earliest structural events in DN, and inflammatory mechanisms may have a significant impact on podocyte integrity and survival. Experimental studies demonstrate that inflammasome activation in podocytes leads to cytoskeletal rearrangement, slit diaphragm dysfunction, release of proinflammatory mediators, and, in advanced stages, pyroptosis, a caspase‐1–dependent inflammatory form of cell death [18].

The intense immunostaining observed in podocytes in our DN samples is consistent with a possible contribution of inflammasome pathways to glomerular deterioration and highlights the potential role of the podocyte as both a target and an amplifier of local inflammation.

Increased expression in glomerular endothelial cells is consistent with the involvement of inflammasome‐mediated injury in the microvascular component of DN. Interactions between AGEs and RAGE, enhanced ROS generation, and upregulation of adhesion molecules such as ICAM‐1 and VCAM‐1 create a proinflammatory endothelial environment conducive to NLRP3 activation. Endothelial activation, in turn, may facilitate leukocyte recruitment, potentially amplifying glomerular injury, and may increase vascular permeability, reinforcing the centrality of endothelial dysfunction in DN.

The marked interstitial expression of NLRP3 and IL‐1β and their correlation with serum creatinine levels suggest a possible association with progressive renal functional decline. Although DN has historically been approached as a glomerular disease, accumulating evidence indicates that the extent of interstitial inflammation and fibrosis is a stronger predictor of GFR decline than glomerular injury alone [14]. The correlations observed in this study support the potential relevance of the interstitial compartment in the pathophysiology of DN and suggest that inflammasome activation in this region may contribute to renal functional decline. However, these findings should be interpreted as exploratory rather than definitive.

The behavior of IL‐18 warrants additional consideration. Although, like IL‐1β, it is a direct product of caspase‐1 activation, IL‐18 showed a more heterogeneous pattern, with prominent expression in podocytes and a trend toward increased interstitial expression. This variability may reflect intrinsic differences in cytokine regulation or microenvironmental characteristics within the kidney. IL‐18 is known to promote endothelial adhesion molecule expression, enhance leukocyte recruitment, and stimulate fibrogenic processes, particularly in intermediate and advanced stages of DN [20]. Its high expression in podocytes may indicate earlier activation of glomerular inflammatory pathways, whereas its variable interstitial expression may reflect differing degrees of tissue remodeling among patients.

Another important aspect involves integrating immunohistochemical findings with clinical characteristics. Most patients in this study had long‐standing diabetes, a high prevalence of systemic hypertension, and marked proteinuria—factors that are well known to exacerbate renal inflammation. The predominance of class III and IV cases according to Tervaert et al. [23] further indicates that many patients were in advanced stages of DN, which may be associated with increased inflammasome activation; however, the cross‐sectional design does not allow assessment of temporal relationships or progression of renal dysfunction. Contemporary studies suggest that inflammasome activity is modulated by hyperglycemia severity, mitochondrial dysfunction, and concomitant hypertension [25, 26].

Although the correlations observed in the bivariate analyses restricted to patients with DN were of modest magnitude, this finding should be interpreted within the context of the multifactorial nature of DN. GFR is influenced by multiple metabolic, hemodynamic, inflammatory, and structural factors; therefore, it would not be expected that the isolated expression of a single tissue marker would account for a large proportion of renal function changes.

Nevertheless, the direction of the observed associations, their predominance within the interstitial/inflammatory infiltrate compartment, and the persistence of the association between NLRP3 and IL‐1β expression and lower GFR after adjustment for systemic arterial hypertension, diabetes duration, and proteinuria support the hypothesis that local activation of the inflammasome pathway is related to renal dysfunction in DN. This interpretation is consistent with previous evidence in the literature implicating the NLRP3–IL‐1β/IL‐18 pathway in renal inflammation and fibrosis in models of diabetic kidney disease.

Therefore, our findings should not be interpreted as proof of causality or as evidence that these markers are isolated determinants of the renal function. Rather, they provide evidence supporting the involvement of the inflammasome pathway in the renal dysfunction associated with DN in human tissues.

These findings raise the possibility that NLRP3, IL‐1β, and IL‐18 may serve as markers of inflammatory activity and tissue damage rather than traditional clinical parameters alone, potentially enabling risk stratification, early identification of accelerated progression, and patient selection for targeted anti‐inflammatory therapies. The NLRP3/IL‐1β axis, in particular, has been increasingly explored as a promising therapeutic target in both type 2 diabetes and inflammatory renal diseases.

NLRP3 inhibitors such as MCC950 and related compounds, as well as IL‐1β blockers, have shown experimental benefits and may eventually form part of therapeutic strategies for DN [25]. The present findings provide additional support for investigating this pathway.

Recent studies have suggested that the modulation of inflammatory pathways, particularly through the inhibition of proinflammatory cytokines, may represent a promising therapeutic strategy for DN and other renal diseases. In this context, natural bioactive compounds have gained increasing attention. Orientin, a flavonoid with anti‐inflammatory and antioxidant properties, has demonstrated the ability to attenuate inflammatory mediators such as IL‐1β, IL‐6, TNF‐α, and TGF‐β1 [27]. Similarly, L‐carvone, a chiral monoterpenoid ketone with relevant immunomodulatory effects, has shown in experimental studies the ability to reduce the expression of inflammatory cytokines, including TNF‐α and IL‐1β [28, 29]. Together, these findings reinforce the potential of inflammatory modulation as a therapeutic target in DN.

In this scenario, the study of central inflammatory pathways such as the NLRP3 inflammasome and its effector cytokines, particularly IL‐1β and IL‐18, becomes especially relevant. Thus, a deeper understanding of the activation of the NLRP3–IL‐1β/IL‐18 axis may not only elucidate the pathophysiological mechanisms involved in DN but also guide the development of more targeted and effective therapeutic strategies.

The study presents some limitations, such as the use of autopsy tissue as controls, the semiquantitative nature of immunohistochemical evaluation, and incomplete availability of some clinical variables. These aspects should be considered when interpreting the findings. Samples were carefully selected to minimize preanalytical variability, analyses were performed in a blinded manner following standardized protocols, and the large number of biopsies evaluated provides robust support for the conclusions. Thus, this study brings together a well‐characterized national cohort of DN biopsies, composed of samples obtained from different regions of Brazil, combined with a systematic assessment of inflammasome‐related mediators across multiple renal compartments. In this context, our findings provide evidence supporting an association between the NLRP3/IL‐1β/IL‐18 axis and both glomerular inflammation and interstitial injury, which, in turn, are related to renal dysfunction. These results add to the existing literature by providing a more comprehensive characterization of the inflammatory pathways involved in DN.

These findings underscore the potential of these mediators as biomarkers of inflammatory activity and support further investigation in longitudinal and mechanistic studies to clarify their prognostic and therapeutic relevance in the management of DN.

## 5. Conclusion

The findings of this study demonstrate that DN is characterized by broad and compartmentalized activation of the NLRP3 inflammasome, accompanied by increased expression of the proinflammatory cytokines IL‐1β and IL‐18 in glomerular cells and within the interstitial compartment. The presence of these mediators across multiple renal structures, together with their correlation with serum creatinine levels, supports an association between inflammation mediated by the NLRP3/IL‐1β/IL‐18 axis and renal injury. These observations suggest that inflammasome activation may represent a relevant component of the inflammatory environment. In this context, the NLRP3/IL‐1β/IL‐18 axis may represent a potential biomarker of inflammatory activity and a candidate therapeutic target, although its prognostic value and therapeutic implications require confirmation in longitudinal and mechanistic studies.

## Funding

The present study received the following funding: CNPq through Call Number 09/2023 – Research Productivity Fellowships (PQ) (Grant 313894/2023‐0) for the project “In situ identification of inflammasome pathways, immune response profiles, and cell death in glomerular diseases” and FAPEMIG through Universal Demand Number 001/2023 (Grant APQ‐02831‐23) for the project “Morphology and Molecular Profile of Glomerular Diseases in Humans and Experimental Models: Identification of Potential Therapeutic Targets and Diagnostic Markers.”

## Conflicts of Interest

The authors declare no conflicts of interest.

## Supporting Information

Additional supporting information can be found online in the Supporting Information section.

## Supporting information


**Supporting Information** Table S1: Multivariable linear regression models evaluating the association between renal expression of NLRP3, IL‐1β, and IL‐18 (in podocytes and inflammatory infiltrates) and estimated glomerular filtration rate (eGFR) in patients with diabetic nephropathy. Models were adjusted for hypertension, diabetes duration, and proteinuria. The table includes regression coefficients (B), standard errors, 95% confidence intervals, *p*‐values, and variance inflation factors (VIFs).

## Data Availability

All relevant data are within the manuscript. Further enquiries can be directed to the corresponding author.

## References

[1] Sagoo M. K. and Gnudi L. , Métodos Em Biologia Molecular, Nefropatia Diabética: Uma Visão Geral. Nefropatia Diabética, 2020, 2067, Springer Nature, 10.1007/978-1-4939-9841-8_1.

[2] Samsu N. , Diabetic Nephropathy: Challenges in Pathogenesis, Diagnosis, and Treatment, BioMed Research International. (2021) 2021, no. 1, 10.1155/2021/1497449, 1497449. 34307650 PMC8285185

[3] Rayego-Mateos S. , Morgado-Pascal J. L. , and Opazo-Ríoz L. , et al.Pathogenic Pathways and Therapeutic Approaches Targeting Inflammation in Diabetic Nephropathy, International Journal of Molecular Sciences. (2020) 21, no. 11, 10.3390/ijms21113798, 3798. 32471207 PMC7312633

[4] Dwivedi S. and Sikarwar M. S. , Diabetic Nephropathy: Pathogenesis, Mechanisms, and Therapeutic Strategies, Hormone and Metabolic Research. (2021) 57, no. 1, 7–17, 10.1055/a-2435-8264. 39572154

[5] Bouça B. , Bogalho A. P. , and Agapito A. , Nefropatia Diabética, Revista Portuguesa de Diabetes. (2021) 16, no. 2, 80–89, https://www.revportdiabetes.com/wp-content/uploads/2021/07/RPD_Junho_2021_ARTIGO-DE-REVISAO_80-89.pdf.

[6] Araújo L. S. , Torquato B. G. S. , and da Silva C. A. , et al.Renal Expression of Cytokines and Chemokines in Diabetic Nephropathy, BMC Nephrology. (2020) 21, 10.1186/s12882-020-01960-0, 308. 32723296 PMC7389446

[7] Bessa F. X. M. , Perfil Epidemiológico Dos Pacientes Com Diabetes Mellitus, Submetidos a Biópsia Renal Em Um Hospital Terciário, Revista Contemporânea. (2024) 4, no. 12, https://ojs.revistacontemporanea.com/ojs/index.php/home/article/view/6983, 6983.

[8] Caviedes-Cleves M. A. , Arias L. F. , and Ospina-Ospina S. , Achados Histopatológicos Em Biópsia Renal De Pacientes Com COVID-19 e Envolvimento Renal, Medicina e Laboratório. (2022) 26, no. 3, 261–271, https://fi-admin.bvsalud.org/document/view/v7ksj.

[9] Wu T. , Ding L. , Andoh V. , Zhang J. , and Chen L. , The Mechanism of Hyperglycemia-Induced Renal Cell Injury in Diabetic Nephropathy Disease: An Update, Life. (2023) 13, no. 2, 10.3390/life13020539, 539. 36836895 PMC9967500

[10] Efiong E. E. , Maedler K. , and Effa E. , et al.Decoding Diabetic Kidney Disease: A Comprehensive Review of Interconnected Pathways, Molecular Mediators, and Therapeutic Insights, Diabetology & Metabolic Syndrome v. (2025) 17, no. 1, 10.1186/s13098-025-01726-4, 192. PMC12139089 40468401

[11] Agarwal R. , Pathogenesis of Diabetic Nephropathy, Chronic Kidney Disease and Type 2 Diabetes, 2021, American Diabetes Association, 10.2337/db20211-2. 34279881

[12] Matoba K. , Takeda Y. , Nagia Y. , Kawanami D. , Utsunomiya K. , and Nishimura R. , Unraveling the Role of Inflammation in the Pathogenesis of Diabetic Kidney Disease, International Journal of Molecular Sciences. (2019) 20, no. 14, 10.3390/ijms20143393, 3393. 31295940 PMC6678414

[13] Lin Y. C. , Chang Y. H. , Yang S. Y. , Wu K. D. , and Chu T. S. , Update of Pathophysiology and Management of Diabetic Kidney Disease, Journal of the Formosan Medical Association. (2018) 117, no. 8, 662–675, 10.1016/j.jfma.2018.02.007. 29486908

[14] Chen J. , Liu Q. , He J. , and Li Y. , Immune Responses in Diabetic Nephropathy: Pathogenic Mechanisms and Therapeutic Target, Frontiers in Immunology. (2022) 13, 10.3389/fimmu.2022.958790, 958790. 36045667 PMC9420855

[15] Komada T. and Muruve D. A. , The Role of Inflammasomes in Kidney Disease, Nature Reviews Nephrology. (2019) 15, no. 8, 501–520, 10.1038/s41581-019-0158-z. 31164720

[16] Tang S. C. W. and Yiu W. H. , Innate Immunity in Diabetic Kidney Disease, Nature Reviews Nephrology. (2020) 16, no. 4, 206–222, 10.1038/s41581-019-0234-4. 31942046

[17] Xiong W. , Meng X.-F. , and Zhang C. , NLRP3 Inflammasome in Metabolic-Associated Kidney Diseases: An Update, Frontiers in Immunology. (2021) 12, 10.3389/fimmu.2021.714340, 714340. 34305953 PMC8297462

[18] Shahzad K. , Fatima S. , and Khawaja H. , et al.Podocyte-Specific Nlrp3 Inflammasome Activation Promotes Diabetic Kidney Disease, Kidney International. (2022) 102, no. 4, 766–779, 10.1016/j.kint.2022.06.010. 35779608

[19] Kovalik M. E. , Dacanay M. A. , Crowley S. D. , and Hall G. , Swollen Feet: Considering the Paradoxical Roles of Interleukins in Nephrotic Syndrome, Biomedicines. (2024) 12, no. 4, 10.3390/biomedicines12040738, 738. 38672094 PMC11048099

[20] Yaribeygi H. , Atkin S. L. , and Sahebkar A. , Interleukin-18 and Diabetic Nephropathy: A Review, Journal of Cellular Physiology. (2018) 234, no. 5, 5674–5682, 10.1002/jcp.27427. 30417374

[21] Ferro A. B. , Controle De Qualidade, Imuno-Histoquímica, 2014, Amadeu Borges Ferro, 99–100, Chapter 12 https://repositorio.ipl.pt/entities/publication/3c461218-22c6-4942-9f7b-25160f1328b7.

[22] Kim S. W. , Roh J. , and Park C. S. , Immunohistochemistry for Pathologists: Protocols, Pitfalls, and Tips, Journal of Pathology and Translational Medicine. (2016) 50, 411–418, 10.4132/jptm.2016.08.08. 27809448 PMC5122731

[23] Tervaert T. W. , Mooyaart A. L. , Amann K. , Cohen A. H. , Higano S. T. , and Fiers T. , Pathologic Classification of Diabetic Nephropathy, Journal of the American Society of Nephrology. (2010) 21, no. 4, 556–563, 10.1681/ASN.2010010010. 20167701

[24] Williams B. M. , Cliff C. L. , Lee K. , Squires P. E. , and Hills C. E. , The Role of the NLRP3 Inflammasome in Mediating Glomerular and Tubular Injury in Diabetic Nephropathy, Frontiers in Physiology. (2022) 13, 10.3389/fphys.2022.907504, 907504. 35755447 PMC9218738

[25] Meier D. T. , de Paula Souza J. , and Donath M. Y. , Targeting the NLRP3 Inflammasome–IL-1β Pathway in Type 2 Diabetes and Obesity, Diabetologia. (2025) 68, no. 1, 3–16, 10.1007/s00125-024-06306-1. 39496966 PMC11663173

[26] Li X. , Liu S. , Huangfu J. , Lai N. , and Shang Y. , Clinical Significance of NLRP3 Inflammasome and Related Cell Molecules in Early Diabetic Kidney Disease in Elderly Population, Journal of Medical Biochemistry. (2024) 43, no. 6, 828–834, 10.5937/jomb0-45950. 39876912 PMC11771978

[27] Luty R. S. , Al-Zubaidy A. A. , Malik A. S. , Ridha-Salman H. , and Abbas A. H. , Protective Effect of Orientin on Diabetic Nephropathy in Rat Models of High-Fat Diet and Streptozotocin-Induced Diabetes, Naunyn-Schmiedeberg’s Archives of Pharmacology. (2025) 398, no. 8, 10769–10784, 10.1007/s00210-025-03949-8. 40035824

[28] Shareef S. M. , Kathem S. H. , and Ridha-Salman H. , L-Carvone Alleviates Lipopolysaccharide-Induced Acute Kidney Injury via Modulating MyD88-Dependent and Independent Signaling Pathways in Mice, Molecular Biology Reports. (2025) 53, no. 1, 10.1007/s11033-025-11213-8, 39. 41175326

[29] Shareef S. M. , Kathem S. H. , and Ridha-Salman H. , Nephroprotective Effect of L-Carvone on a Mouse Model of LPS-Induced Sepsis-Associated Renal Injury via Regulation of TLR4/NF-κB/AP-1/IRF-3 and Nrf2/iNOS Molecular Signaling Cascades, Journal of Taibah University Medical Sciences. (2026) 21, no. 1, 49–66, 10.1016/j.jtumed.2025.12.008. 41585603 PMC12828391

