# Supplementary material for: Tissue Expression of NLRP3, IL‐1β, and IL‐18 in Glomerular and Interstitial Inflammation Associated With Diabetic Nephropathy
Source: Mediators Inflamm. 2026 Aug 6;2026:9937801. doi: 10.1155/mi/9937801 (PMC13446144; doi:10.1155/mi/9937801)
Supplement: Supplementary file 1 [file MI-2026-9937801-s001.docx]

**Supplementary material: Multivariable linear regression models evaluating the association between inflammasome marker expression and eGFR in patients with diabetic nephropathy**

| **Independent variables** | **B** | **Standard Error** | **95% CI** | **p-value** | **VIF** |
| --- | --- | --- | --- | --- | --- |
| **NLRP3 in podocytes** |  |  |  |  |  |
| NLRP3 (%) | -9.778 | 24.751 | -59.764 – 40.208 | 0.695 | 1.030 |
| Proteinuria (mg/24h) | -0.068 | 1.038 | -2.164 – 2.028 | 0.948 | 1.069 |
| Hypertension | -11.179 | 11.622 | -34.649 – 12.291 | 0.342 | 1.085 |
| Diabetes Duration | -7.054 | 10.372 | -28.001 – 13.893 | 0.500 | 1.030 |
|  |  |  |  |  |  |
| **NLRP3 in infiltrate** |  |  |  |  |  |
| NLRP3 (%) | -53.131 | 23.712 | -101.019 – -5.244 | 0.031* | 1.082 |
| Proteinuria (mg/24h) | -0.609 | 1.012 | -2.652 – 1.434 | 0.550 | 1.136 |
| Hypertension | -8.863 | 10.982 | -31.042 – 13.315 | 0.424 | 1.083 |
| Diabetes Duration | -4.335 | 9.835 | -24.197 – 15.526 | 0.662 | 1.035 |
|  |  |  |  |  |  |
| **IL-18 in podocytes** |  |  |  |  |  |
| IL-18 (%) | 35.795 | 62.252 | -89.925 – 161.515 | 0.568 | 1.046 |
| Proteinuria (mg/24h) | -0.002 | 1.039 | -2.100 – 2.096 | 0.999 | 1.076 |
| Hypertension | -11.644 | 11.651 | -35.173 – 11.884 | 0.323 | 1.095 |
| Diabetes Duration | -8.617 | 10.722 | -29.664 – 12.430 | 0.413 | 1.044 |
|  |  |  |  |  |  |
| **IL-18 in infiltrate** |  |  |  |  |  |
| IL-18 (%) | -50.793 | 41.331 | -134.263 – 32.676 | 0.226 | 1.044 |
| Proteinuria (mg/24h) | -0.182 | 1.026 | -2.253 – 1.890 | 0.860 | 1.079 |
| Hypertension | -7.941 | 11.624 | -31.415 – 15.533 | 0.498 | 1.121 |
| Diabetes Duration | -7.128 | 10.128 | -27.581 – 13.326 | 0.486 | 1.074 |
|  |  |  |  |  |  |
| **IL-1β in podocytes** |  |  |  |  |  |
| IL-1β (%) | -30.282 | 28.772 | -88.389 – 27.825 | 0.299 | 1.078 |
| Proteinuria (mg/24h) | -0.221 | 1.038 | -2.316 – 1.875 | 0.833 | 1.094 |
| Hypertension | -12.222 | 11.528 | -35.503 – 11.059 | 0.295 | 1.092 |
| Diabetes Duration | -5.891 | 10.295 | -26.683 – 14.900 | 0.570 | 1.038 |
|  |  |  |  |  |  |
| **IL-1β in infiltrate** |  |  |  |  |  |
| IL-1β (%) | -73.940 | 33.036 | -140.658 – -7.223 | 0.031* | 1.056 |
| Proteinuria (mg/24h) | -0.101 | 0.981 | -2.082 – 1.880 | 0.918 | 1.068 |
| Hypertension | -6.404 | 11.123 | -28.868 – 16.059 | 0.568 | 1.111 |
| Diabetes Duration | -10.480 | 9.815 | -30.301 – 9.340 | 0.292 | 1.031 |

eGFR (estimated glomerular filtration rate) was used as the dependent variable. Each inflammasome marker was evaluated in a separate model according to renal compartment. Models were adjusted for hypertension, diabetes duration, and proteinuria. B coefficients represent the estimated change in eGFR associated with a one-unit increase in marker expression, holding the other covariates constant. VIF values were used to assess multicollinearity among predictors. Due to missing clinical data, multivariable analyses were performed using complete cases only (n = 46). VIF: variance inflation factor. *p < 0.05.
